# Supplementary material for: Tracking daily activities with ecological momentary assessment: A bibliometric analysis of current use in health Mapping daily activities with ecological momentary assessment
Source: PLOS Digit Health. 2026 Mar 31;5(3):e0001331. doi: 10.1371/journal.pdig.0001331 (PMC13037968; doi:10.1371/journal.pdig.0001331)
Supplement: Attachment — Submitted filename: Respons to reviewers .docx [file pdig.0001331.s003.docx]

**Rebuttal letter: Tracking daily activities with ecological momentary assessment: a bibliometric analysis of current use in health. Mapping daily activities with ecological momentary assessment.**

Dear Editor and reviewer

Thank you for your thorough and thoughtful feedback on our manuscript. We appreciate the

time and effort you have dedicated to improving our work, and we trust that the implemented changes meet your satisfaction. Below, we provide detailed, point-by-point responses to each of your comments.

Kind regards

Eva Delooz

prof. dr. Bruno Bonnechère

prof. dr. Barbara Piškur

prof. dr. Annemie Spooren

**Journal Requirements**

1. Please amend your detailed Financial Disclosure statement. This is published with the article. It must therefore be completed in full sentences and contain the exact wording you wish to be published.

1. Please clarify all sources of funding (financial or material support) for your study. List the grants (with grant number) or organizations (with url) that supported your study, including funding received from your institution.

2. State the initials, alongside each funding source, of each author to receive each grant.

3. State what role the funders took in the study. If the funders had no role in your study, please state: “The funders had no role in study design, data collection and analysis, decision to publish, or preparation of the manuscript.”

4. If any authors received a salary from any of your funders, please state which authors and which funders.

We have updated the Financial Disclosure section in the submission system to clearly specify the funding source, grant number, recipient author and role of the funder.

*Financial disclosure section: This research was supported by a doctoral research grant from Hasselt University awarded to the principal investigator (Eva Delooz), grant number BOF24DOC43. No other financial or material support was received for this study. The funder had no role in study design, data collection and analysis, decision to publish, or preparation of the manuscript. No authors received a salary or other financial compensation specifically for the conduct of this study.*

2. We have amended your Competing Interest statement to comply with journal style. We kindly ask that you double check the statement and let us know if anything is incorrect.

We have reviewed the amended Competing Interests statement and confirm that it is correct. The authors declare no competing interests.

3. In the online submission form, you indicated that the data underlying this study are available from the Web of Science database (Clarivate Analytics), subject to their access and licensing terms. The dataset analyzed during the current study, including records of 3,692 articles, and the R scripts used for data processing and analysis, are available from the corresponding author upon reasonable request.

All PLOS journals now require all data underlying the findings described in their manuscript to be freely available to other researchers, either
1. In a public repository,
2. Within the manuscript itself, or
3. Uploaded as supplementary information.

To fully comply with the journal’s data sharing policy, we have now made the materials underlying this study publicly available via Zenodo. Specifically, we have deposited: (i) the R scripts used for data processing and analysis, (ii) the R version and package information, (iii) the full search strategy and search terms, and (iv) a detailed README file with step-by-step instructions to reproduce the analyses. Due to licensing restrictions of the Web of Science (Clarivate Analytics), the raw bibliographic records (n = 3,692 articles) cannot be shared publicly. However, all information necessary to reproduce the data collection process from Web of Science is provided in the repository. Researchers with access to Web of Science can therefore fully reproduce the dataset following the documented search strategy. The Zenodo repository is publicly accessible at <https://doi.org/10.5281/zenodo.18163871>.

*Added lines in the manuscript: lines 201-205: All R scripts, search terms, and documentation necessary to reproduce the analyses, including R version information (R version 2024.12.0+467), are available in a public Zenodo repository with DOI* [*10.5281/zenodo.18163870*](https://doi.org/10.5281/zenodo.18163870)*. The underlying bibliographic data were obtained from Web of Science (Clarivate Analytics) and cannot be shared publicly due to licensing restrictions.*

*Adapted data availability statement: All materials necessary to reproduce the analyses are publicly available via Zenodo (DOI: 10.5281/zenodo.18163870). The repository includes (i) all R scripts used for data processing and analysis, (ii) detailed R version and package information (R version 2024.12.0+467), (iii) the complete search strategy and search terms, and (iv) a comprehensive README file with step-by-step instructions to reproduce the analyses. Due to licensing restrictions imposed by Web of Science (Clarivate Analytics), the raw bibliographic records underlying this study (n = 3,692 articles) cannot be shared publicly. However, all information required to reproduce the data collection process from Web of Science is provided in the repository. Researchers with institutional access to Web of Science can therefore fully reproduce the dataset by following the documented search strategy.*

4. Please provide separate figure files in .tif or .eps format.

For more information about figure files please see our guidelines:
<https://journals.plos.org/digitalhealth/s/figures>
<https://journals.plos.org/digitalhealth/s/figures#loc-file-requirements>

All figures are now provided as separate files in .tif format, in accordance with the PLOS figure guidelines.

5. We have noticed that you have uploaded Supporting Information files, but you have not included a list of legends. Please add a full list of legends for your Supporting Information files after the references list.

We have added a Supporting Information section after the references, providing a complete list of legends for all uploaded Supporting Information files, including the bibliometric checklist.

*Added lines in the manuscript: lines 532-535: A.*  *The BIBLIO checklist for reporting the bibliometric reviews of the biomedical literature. Checklist reporting the methodological details of the bibliometric analysis conducted in this study.*

**Editor Comments:**

6. PLOS Digital Health requires open data deposit with a permanent identifier. Your current statement, “available from the corresponding author upon reasonable request”, does not meet journal policy. Upload your processed dataset or script to replicate data extraction (not raw Web of Science exports, which are proprietary), term lists, and R scripts to a suitable data deposit. Provide the DOI in your Data Availability Statement. Include R package versions for reproducibility.

To fully comply with the journal’s data sharing policy, we have now made the materials underlying this study publicly available via Zenodo (see also comment 3 on data availability statement). Specifically, we have deposited: (i) the R scripts used for data processing and analysis, (ii) the R version and package information, (iii) the full search strategy and search terms, and (iv) a detailed README file with step-by-step instructions to reproduce the analyses. Due to licensing restrictions of the Web of Science (Clarivate Analytics), the raw bibliographic records (n = 3,692 articles) cannot be shared publicly. However, all information necessary to reproduce the data collection process from Web of Science is provided in the repository. Researchers with access to Web of Science can therefore fully reproduce the dataset following the documented search strategy. The Zenodo repository is publicly accessible at [https://doi.org/10.5281/zenodo.1816387](https://doi.org/10.5281/zenodo.18163871)0 (see also answer on comment 3, added lines in manuscript:lines 201-205)

7. Your analysis classified publications using keyword matching of titles, abstracts, and metadata. As I understand you did not examine actual EMA protocols or study designs. You describe this approach Lines 324-329 describe this approach, but the classification process needs fuller methodological detail. The methods mention using an R 'list' function with keywords in supplementary materials, but several specifics remain unclear: Which bibliographic fields did you search (title only, or title + abstract + keywords)? Could one publication be assigned to multiple categories? Was classification purely automated, or did you manually verify any assignments? If purely algorithmic, acknowledge that keyword matching cannot capture nuanced content. A paper discussing depression and physical activity in its results section might be misclassified if those terms appear only there, not in the title or abstract you searched.

We have now expanded the Methods section to more explicitly describe the bibliographic fields used, the nature of the classification, the handling of multiple potential categories, and the iterative refinement of the keyword lists. Publications were classified using a keyword-matching procedure applied to multiple bibliographic fields (title, abstract, author keywords, Keywords Plus, subject categories, and Web of Science categories). Each publication was assigned to a single category based on the first matching category detected. Multiple category assignments were therefore not allowed. The classification was fully algorithmic and no manual reassignment of individual publications was performed. However, the keyword lists were iteratively refined during analysis by examining unclassified records and expanding the keyword lists where conceptually appropriate, after which the classification procedure was rerun. We now explicitly acknowledge that the keyword-matching procedure is inherently limited in capturing nuanced or implicit content that appears outside searchable bibliographic fields (e.g., results sections not indexed in abstracts), and that some degree of misclassification cannot be excluded. This limitation has been added to the discussion section (lines 393-401). This approach is consistent with the broader methodological strategy commonly used in a bibliometric study, which prioritized reproducibility and scalability over manual screening (see also answer to comment 8, added lines in manuscript: lines 143-145 and answer to comment 12, added figure 2).

*Added lines in the manuscript: lines 186-198: The classification of the publications throughout the different components, relied on a keyword-matching procedure implemented in R. Predefined lists of stemmed keywords were matched against multiple bibliographic fields, including titles (TI), abstracts (AB), author keywords (DE), Keywords Plus (ID), Web of Science categories, and subject categories. These fields were concatenated into a single searchable text string for each publication. Each publication was assigned to a single category based on the first detected keyword match. Multiple category assignments were therefore not permitted. If no match was identified in the combined fields, additional matching was performed sequentially on author keywords and titles only. The classification process was fully algorithmic, and individual publications were not manually reassigned. To improve coverage, the keyword lists were iteratively refined by inspecting records that remained unclassified and identifying recurring relevant terms, after which the classification procedure was rerun until no further systematic assignments could be made.*

*Adapted lines in the manuscript: lines 393-401: Methodologically, the classification of publications into disability and health domains, as well as PEO components, relied on a keyword-matching procedure implemented in R. While this approach ensured systematic and reproducible categorization across a large corpus of publications, the resulting classifications were inherently shaped by the predefined keyword lists and the availability of information within indexed bibliographic fields (e.g., titles, abstracts, and metadata). Consequently, publications in which relevant concepts were discussed primarily outside these fields may not have been fully captured. To enhance transparency and reproducibility, the complete list of search terms and classification criteria is provided in the supplementary material.*

8. Your search was limited to Web of Science using full phrases ("ecological momentary assessment," "experience sampling") but excluded the acronyms EMA and ESM. Many EMA papers use only acronyms in titles and abstracts, creating risk of missed relevant studies. I recognize these abbreviations appear in other fields, which may have deterred their inclusion. However, a pilot search combining acronyms with your EMA-specific terms could quantify this trade-off: test whether "EMA AND (daily activities OR functioning)" captures additional relevant papers or merely increases noise. Your own data demonstrate the cost of overly broad terms, 201 irrelevant papers from energy and computer science entered your initial results. The limitations section should acknowledge that excluding acronyms prioritized precision over sensitivity, and that the true scope of EMA literature on daily activities may be larger than your dataset suggests.

We agree that excluding the acronyms “EMA” and “ESM” may have reduced the sensitivity of the search strategy. However, during an initial scoping phase of search development, we observed that including these acronyms resulted in substantial noise, with a large number of retrieved records using the same letter combinations to denote fully unrelated concepts. To minimize the inclusion of non-relevant literature and ensure a manageable and conceptually coherent dataset, we therefore deliberately restricted the search to the full phrases “ecological momentary assessment” and “experience sampling.” This decision reflects a methodological trade-off prioritizing precision over sensitivity. The keyword-matching procedure was performed not only on the title but also on the abstract, where, in most cases, the terms are first described in full. Although it is possible that a small number of relevant studies using acronyms exclusively may have been missed, this number is expected to be limited. We therefore consider that the potential loss of sensitivity was limited and outweighed by the reduction in non-relevant records. While the true scope of EMA-based research on daily activities and functioning may be slightly broader than represented here, this is unlikely to have affected the overall conclusions.

*Added lines in the manuscript: lines 143-145: These search terms were refined during an initial scoping phase to balance sensitivity and precision to improve the quality of the search.*

*Added lines in the manuscript: lines 371-377: To reduce false positives and maintain a conceptually coherent dataset, the search strategy prioritized precision by restricting queries to the full phrases “ecological momentary assessment” and “experience sampling”, rather than the use of acronyms such as EMA or ESM. As a result, some relevant studies using acronyms exclusively may not have been captured; however, this number is expected to be small, as these terms are typically defined in full in abstracts. Consequently, the total volume of EMA research on daily activities and functioning may be slightly underestimated.*

9. The Venn diagrams visually suggest quantitative proportions but provide no scale or percentages. Either add actual values to each region showing overlap between person, occupation, and environment, or state clearly in the caption that the diagrams are conceptual schematics not drawn to scale.

The Venn diagrams were intended as conceptual schematics to illustrate the theoretical relationships between person, occupation, and environment, rather than to represent quantitative proportions.

*Added lines in the manuscript: lines 253-254: While numerical distributions are reported in the text, Figure 5a and 5b serves solely as a conceptual visualization and does not depict relative magnitudes.*

**Reviewer 1**

10. Comment 1: The stated objectives and methodology are partly aligned, as the objective aims to "systematically map the use of EMA to understand lived health" but the methods only perform keyword-based classification of publications rather than analyzing actual EMA applications.

We agree that our methodology does not analyze individual EMA/ESM apps, protocols or study designs, but instead applies a bibliometric approach to systematically map the published literature of the current body or evidence. To avoid any ambiguity, we have refined the study aim to explicitly refer to the mapping of published EMA research rather than the analysis of EMA applications themselves. This revision ensures full alignment between the stated objectives and the methodological approach.

*Adapted lines in the manuscript: line 131: A bibliometric analysis was conducted to systematically map the published literature on EMA use in studying lived health and daily activities in disability and healthy populations.*

11. Comment 2: The methodology is not clearly and completely reported. The objectives, measures and unit of analysis and the analysis details are incompletely reported. Also, Which component of R package was used is not mentioned. In addition, why the database WoS selected needs justification.

First, we now explicitly define the unit of analysis as individual bibliographic records (i.e., single publications) retrieved from the Web of Science database. Second, we have clarified the objectives, measures, and analytical procedures, specifying the bibliographic fields used (titles, abstracts, author keywords, Keywords Plus, subject categories, and Web of Science categories), the automated nature of the keyword-matching procedure, and the handling of multiple potential category matches. The full classification workflow is now described step-by-step, including the iterative refinement of keyword lists and the decision rules applied during classification. Third, we now explicitly report the software environment and R packages used, including their functional roles. Data import and bibliometric structuring were performed using bib2df and bibliometrix; data wrangling relied on the tidyverseecosystem (including dplyr, tidyr, forcats, and purrr); text preprocessing and keyword-matching procedure were implemented using stringr and SnowballC; and visualizations were generated using ggplot2, ggforce, DiagrammeR, DiagrammeRsvg, magick, and maps. This information was added in the [read.md](http://read.md) file at [10.5281/zenodo.18163870](https://doi.org/10.5281/zenodo.18163870). Fourth, we now provide an explicit justification for the selection of Web of Science as the source database. Web of Science was chosen due to its standardized and well-structured bibliographic fields, availability of Keywords Plus and subject categories, and suitability for large-scale bibliometric analyses requiring consistent metadata across records (see also answer to comment 7, added lines in manuscript: lines 186-196 and lines 393-401). Finally, to further enhance transparency and reproducibility, all R scripts, search strategies, package information, and detailed documentation have been made publicly available via Zenodo. While raw bibliographic records cannot be shared due to licensing restrictions, all information required to fully reproduce the data collection and analysis is provided (see also answer to comment 3, added lines in manuscript: lines 201-205).

*Added lines in the manuscript: lines 145-147: Web of Science was selected as the source database due to its standardized bibliographic structure, inclusion of Keywords Plus and subject categories, and suitability for reproducible bibliometric analyses across large publication corpora.*

12. Comment 3: No manual screening process was implemented to verify relevance of the 3,692 included publications, and the post-hoc exclusion of 343 papers (9.3% of the dataset) for "energy sustainability" and "computer science" topics confirms that irrelevant content was initially included.

The absence of a manual title–abstract screening step reflects a deliberate methodological choice consistent with the bibliometric and large-scale nature of this study. Our objective was to systematically map publication patterns and thematic emphases in the EMA literature using reproducible procedures, rather than to conduct a study-level content evaluation of individual EMA protocols or designs. As described in the revised Methods section (see also answer to comment 8, added lines in manuscript: lines 143-145 and answer to comment 7, added lines in manuscript: lines 186-196 and lines 393-401), publications were classified using a keyword-matching procedure applied to multiple bibliographic fields. During this thematic classification process, a subset of publications was identified as unrelated to EMA applications in health and daily functioning—primarily originating from energy sustainability and computer science contexts. These records (n = 343; 9.3% of the initially retrieved dataset) were therefore not retained for subsequent analyses as a direct result of the analytical workflow, rather than through selective or post hoc manual screening . A detailed description of this phased analytical specification is provided in response to Comment 21. To enhance transparency, we have updated the study flowchart to explicitly illustrate this analytical filtering step and the resulting exclusion of irrelevant records. Importantly, all thematic classifications and subsequent analyses were conducted exclusively on the curated dataset comprising publications relevant to EMA applications in health, disability, and daily activities.

*Adapted Figure 2:*

13. Comment 4: The inclusion of irrelevant publications and subsequent selective exclusion raises serious concerns about the validity of all reported findings, as the classification of health categories and PEO components may be systematically biased by contaminated data.

As also described in responses to comments 7, 8, and 12 (see also answer to comment 8, added lines in manuscript: lines 143-145 and answer to comment 7, added lines in manuscript: lines 186-196 and lines 393-401 and answer to comment 12, adapted figure 2), the study employed a Keyword- - matching procedure with iterative refinement of keyword lists and transparent decision rules. The trade-off between sensitivity and precision, including the deliberate exclusion of acronyms and unrelated domains, was carefully managed to maximize conceptual coherence and minimize contamination of the analyzed dataset. We now explicitly acknowledge this trade-off in the Methods and Limitations sections. Overall, the validity of the reported findings is supported by the staged analytical approach adopted in this study. General descriptive analyses (e.g., publication volume and country-level patterns) were conducted on the full, sensitivity-oriented dataset, while domain-specific analyses focusing on health, disability, and PEO components were performed on analytically specified subsets of publications relevant to EMA applications in daily functioning contexts. This phased approach is consistent with standard bibliometric practice and is transparently documented in the revised Methods section and study flowchart (see also answer to comment 12, adapted figure 2 and answer to comment 21).

14. Comment 5: The analysis consists only of descriptive keyword frequencies and basic percentages without employing any advanced bibliometric methods.

Our study employed a "content" bibliometric approach focused on keyword frequencies, thematic categorization, and basic publication trends. While other bibliometric methods (e.g., network analyses or co-citation mapping) were considered in preliminary pilot analyses, these approaches did not directly address our primary research objective: to systematically map the published literature on EMA use in studying lived health and daily activities in disability and healthy populations, and to identify overarching thematic clusters, methodological approaches, and gaps. The descriptive, keyword-matching approach allowed us to quantitatively evaluate publication trends and domain-specific emphases across a large corpus of publications, providing a clear, reproducible, and interpretable overview aligned with the study aims. We explicitly acknowledge that other/advanced bibliometric techniques could provide complementary insights, but the chosen approach was selected to maximize conceptual clarity, reproducibility, and direct relevance to our research questions.

15. Comment 6: The discussion on practical implications of the findings needs to be added.

We have addressed this by expanding the Discussion section to explicitly articulate the practical implications of our findings. Specifically, we added text highlighting how the observed imbalances in EMA research can inform the design of future EMA protocols, enhance ecological validity, and support clinical and rehabilitation practice by better capturing daily activities and contextual factors.

*Added lines in the manuscript: lines 351-361: Beyond its conceptual contributions, these findings have important practical implications. The observed dominance of person-focused EMA studies suggests that current EMA protocols may insufficiently capture the occupational and environmental dimensions of daily functioning. For researchers and clinicians, this highlights the need to design EMA studies that more deliberately integrate activity-related and contextual variables to improve ecological validity and clinical relevance. Incorporating EMA data on daily activities, routines, and environmental context may support more personalized and context-sensitive interventions, particularly in rehabilitation and chronic care settings. Moreover, the identified underrepresentation of EMA applications in several disability and health domains suggests opportunities for broader implementation of EMA to inform practice in populations where daily functioning is substantially affected.*

**Reviewer 2:**

16. Which module was used for the calculations in R?

In our R-based workflow, the analyses were conducted using custom, script-based procedures rather than predefined software modules. Specifically, calculations and classifications were implemented using standard R packages for data handling and text processing, combined with custom-written R scripts that operationalized the stepwise analytical components (general characteristics, disability and health classification, PEO-based categorization, and interaction analysis). To enhance transparency and reproducibility, the complete R script used for the analysis—including all package dependencies and classification procedures—is publicly available via the following repository: <https://doi.org/10.5281/zenodo.18163871>. We have added this information to the Methods section to clarify the analytical workflow (see also answer on comment 3: added lines in manuscript: lines 201-205).

17. Was other software considered for the bibliometric analysis?

R (version 2024.12.0+467) was selected as the primary and sole analytical environment for this study. This choice was made deliberately, as R provides a flexible, transparent, and reproducible framework that allowed us to implement customized, stepwise analytical procedures aligned with the study’s conceptual models (i.e., the PEO framework and the Global Burden of Disease classification) and the research objectives (see also answer on comment 3: added lines in manuscript: lines 201-205). While dedicated bibliometric software packages (e.g., VOSviewer or CiteSpace) are commonly used for network visualization and citation-based analyses, the present study required extensive text-based classification, rule-based filtering, and iterative exclusion across multiple analytical components. These procedures were more efficiently and transparently implemented using R scripts, enabling full control over data preprocessing, classification logic, and reproducibility.

18. The discussion does not sufficiently take into account existing work (including the introduction). Here are some examples:

(1) <https://www.frontiersin.org/journals/psychiatry/articles/10.3389/fpsyt.2024.1300739/full>

(2) <https://www.mdpi.com/1424-8220/24/2/472>

In response to this comment, we have strengthened the integration of existing EMA literature throughout the manuscript. First, we have expanded the Introduction to more clearly position the present study in relation to existing EMA reviews, which predominantly focus on symptom monitoring, methodological characteristics, and technology-driven developments. Second, we have revised the Discussion to explicitly relate our findings to recent bibliometric analyses documenting the rapid growth of EMA research, particularly within mental health contexts and with increasing use of mobile and sensor-based technologies. By doing so, we clarify how our results align with these earlier observations, while also extending existing work by shifting the focus from symptoms and technologies toward daily activities, functioning, and lived health, explicitly framed through the PEO model. These additions improve conceptual continuity between the introduction, discussion, and the existing literature highlighted by the reviewer.

*Added lines in the manuscript: lines 113-118: Recent reviews have examined the use of EMA in health research, with a primary focus on mental health applications, mobile-based interventions, and methodological characteristics such as sampling strategies and compliance (24, 25). While these studies provide valuable overviews of EMA adoption and technological development, they predominantly conceptualize functioning in terms of symptoms, diagnoses, or intervention outcomes.*

*Adapted lines in the manuscript: lines 305-313: This imbalance reflects the historical roots of EMA within psychology, where it was originally developed to monitor symptoms, affective states, and behavioral triggers in real time (16). Importantly, this pattern is consistent with previous bibliometric analyses, which have documented a rapid growth of EMA publications over the past decade, largely driven by mental health research and accompanied by increasing integration of mobile and sensor-based technologies (24). While this focus aligns with dominant EMA research trajectories, it simultaneously highlights a substantial underutilization of EMA in neurological contexts—particularly for capturing engagement in daily activities and functioning.*

19. “Typical” bibliometric analyses also include aspects such as countries, organizations, etc. Why were these not taken into account?

Descriptive information on publication output by country and related characteristics is reported in the Results section (lines 210–222). These analyses provide contextual background on the geographical distribution and growth of EMA research. However, we did not expand this component with additional bibliometric visualizations (e.g., country collaboration networks, institutional co-authorship maps, or citation network analyses). This was a deliberate choice, as the primary aim of this study was to maximize conceptual clarity and alignment with the study’s theoretical framework and objectives. While such analyses are common in bibliometric studies, our primary objective was not to map scientific collaboration structures. In preliminary pilot analyses, we explored additional bibliometric techniques (e.g., network and mapping approaches), but these did not provide direct insight into the conceptual and thematic focus of EMA applications in relation to disability and health categories and daily activities. We therefore adopted a content-oriented bibliometric approach, prioritizing keyword-matching procedure and descriptive trends that directly addressed the research questions.

20. Should patient-reported outcome measures not be included in the search or mentioned as a limitation?

We agree that patient-reported outcome measures (PROMs) are closely related to ecological momentary assessment (EMA) approaches and represent an important body of literature in the assessment of lived health and functioning. In the present study, the search strategy was intentionally restricted to EMA and closely related real-time and ambulatory assessment methodologies. This decision was made to preserve conceptual and methodological coherence, as EMA is characterized by repeated, in-the-moment assessments in naturalistic contexts, which differs from many PROMs that are typically administered retrospectively and at fixed time points. We acknowledge that relevant studies using PROMs without explicitly identifying as EMA may not have been captured by our search strategy. In response to this comment, we have explicitly addressed this issue as a limitation in the manuscript, noting that the exclusion of PROM-focused terminology may have led to an underrepresentation of certain approaches to patient-reported lived health and daily functioning.

*Added lines to the manuscript - lines 377-386: In addition, although patient-reported outcome measures (PROMs) are conceptually related to EMA, they were not explicitly included as search terms. The present study intentionally focuses on EMA-based approaches, defined by repeated, real-time assessments conducted in naturalistic contexts—key methodological characteristics that are not necessarily or explicitly present in PROM-based research. While PROMs constitute an important body of literature in the assessment of lived health and functioning, they represent a distinct methodological tradition. Consequently, the findings specifically reflect trends within EMA-labeled research rather than patient-reported assessment approaches more broadly.*

21. Did any work have to be excluded from the search?

As also described in responses to comments 7, 8, 12 and 13 (see also answer to comment 8, added lines in manuscript: lines 143-145 and answer to comment 7, added lines in manuscript: lines 186-196 and lines 393-401 and answer to comment 12, adapted figure 2) , no publications were excluded from the initial search results. The search strategy was intentionally broad and sensitivity-oriented, and the full set of retrieved records was retained for the initial descriptive and bibliometric analyses (e.g., publication volume and country-level patterns), as illustrated in the adapted study flowchart (Adapted Figure 2). In subsequent analytical stages, the dataset was progressively specified in line with the study’s research questions. During the keyword-matching procedure (Methods, lines 176–188), the analytical focus was narrowed to EMA applications explicitly situated within health and disability contexts. As a result of this, a subset of records (n = 201) originating primarily from energy sustainability and computer science domains fell outside the scope of the health- and disability-focused analyses and was therefore not included in these analyses. A further analytical refinement was applied on the PEO components, which resulted in not including an additional subset of publications (n = 142). Importantly, these steps represent staged analytical refinements rather than post hoc or selective exclusions. No manual title–abstract screening or subjective relevance judgments were applied at any stage. This phased analytical approach allowed us to transparently report general publication patterns based on the full dataset, while ensuring conceptual coherence and relevance in the more focused health-, disability-, and PEO-oriented analyses.
