# Supplementary material for: Tracking daily activities with ecological momentary assessment: A bibliometric analysis of current use in health Mapping daily activities with ecological momentary assessment
Source: PLOS Digit Health. 2026 Mar 31;5(3):e0001331. doi: 10.1371/journal.pdig.0001331 (PMC13037968; doi:10.1371/journal.pdig.0001331)
Supplement: Attachment — Submitted filename: Respons 2 to reviewers .docx [file pdig.0001331.s004.docx]

**Rebuttal letter revision 2: Tracking daily activities with ecological momentary assessment: a bibliometric analysis of current use in health. Mapping daily activities with ecological momentary assessment.**

Dear Editor and reviewer

Thank you for your thorough and thoughtful feedback on our manuscript. We appreciate the

time and effort you have dedicated to improving our work, and we trust that the implemented changes meet your satisfaction. Below, we provide detailed, point-by-point responses to each of your comments.

Kind regards

Eva Delooz

prof. dr. Bruno Bonnechère

prof. dr. Barbara Piškur

prof. dr. Annemie Spooren

1. In the Response to Reviewers (Comment 12 and 21), you state that 343 publications (9.3%) were identified as irrelevant and excluded during the analytical filtering process. However, Figure 2 (Flowchart), the Results text (Section 3.2), and Figure 3 all indicate that 201 publications were excluded for being outside the core scope of disability and health.

Please verify the correct number.

The search strategy was intentionally broad and sensitivity-oriented, and the full dataset (n = 3,692) was retained for the initial descriptive and bibliometric analyses (Stage 1: analysis of publication output and growth rate), as illustrated in the flowchart (Figure 2).

The subsequent analyses followed a four-stage analytical workflow, during which exclusions occurred only at Stages 2 and 3:

- Stage 2: Analysis of disability and health categories (n = 3,491): During the keyword-matching procedure (Methods, lines 176–188), the analytical focus was narrowed to EMA applications explicitly situated within health and disability contexts. As a result, 201 publications—primarily originating from energy sustainability and computer science domains—fell outside the core scope of the disability and health analysis and were therefore not included in subsequent stages.
- Stage 3: Analysis of PEO components (n = 3,349): A further analytical refinement based on the PEO framework resulted in the non-inclusion of an additional 142 publications that did not meet the conceptual requirements of this model.
- Stage 4: Analysis of interactions (n = 3,349): This final analytical stage was conducted on the dataset resulting from Stage 3, with no further exclusions applied.

Taken together, the exclusions at Stage 2 (n = 201) and Stage 3 (n = 142) account for the total of 343 publications (9.3% of the initially retrieved dataset) referenced in our responses to Comments 12 and 21 (revision 1). Importantly, these exclusions reflect predefined, staged analytical specifications rather than post hoc or selective screening. No manual title–abstract screening or subjective relevance judgments were applied at any stage.

2. And one minor formatting issue: In the "Results of the PEO Components" section, the text now refers to Figure 4 , but the conceptual Venn diagrams are labeled as Figure 5.

We have corrected the figure references in the “Results of the PEO Components” section to clearly distinguish between Figure 4, which presents the quantitative distribution of PEO components, and Figure 5a–b, which provides a conceptual (non-quantitative) visualization of the PEO framework. This has been revised to ensure consistent and unambiguous figure referencing throughout the manuscript.

Adapted lines in the manuscript 244-248: The results focusing on 3,349 publications reveal a clear imbalance in how EMA is applied within the PEO framework. As shown in Figure 4, person-oriented research dominates the field, with 2,254 publications (61.1%), compared to substantially fewer studies addressing occupation (659 publications, 17.8%) and environment (321 publications, 8.7%).
